# The Role of High-Resolution Analytical Techniques in the Development of Functional Foods

**DOI:** 10.3390/ijms22063220

**Published:** 2021-03-22

**Authors:** Álvaro Fernández-Ochoa, Francisco Javier Leyva-Jiménez, María De la Luz Cádiz-Gurrea, Sandra Pimentel-Moral, Antonio Segura-Carretero

**Affiliations:** 1 Max Delbrück Center for Molecular Medicine in the Helmholtz Association, 13125 Berlin, Germany; 2 Berlin Institute of Health Metabolomics Platform, 10178 Berlin, Germany; 3 Functional Food Research and Development Center, Health Science Technological Park, Avenida del Conocimiento s/n, E-18100 Granada, Spain; jleyva@cidaf.es (F.J.L.-J.); ansegura@ugr.es (A.S.-C.); 4 Department of Analytical Chemistry, Faculty of Sciences, University of Granada, Fuentenueva s/n, E-18071 Granada, Spain; spimentel@ugr.es

**Keywords:** analytical techniques, analytical chemistry, chromatography, functional food, mass spectrometry, nuclear magnetic resonance, metabolomics, phytochemicals

## Abstract

The approaches based on high-resolution analytical techniques, such as nuclear magnetic resonance or mass spectrometry coupled to chromatographic techniques, have a determining role in several of the stages necessary for the development of functional foods. The analyses of botanical extracts rich in bioactive compounds is one of the fundamental steps in order to identify and quantify their phytochemical composition. However, the compounds characterized in the extracts are not always responsible for the bioactive properties because they generally undergo metabolic reactions before reaching the therapeutic targets. For this reason, analytical techniques are also applied to analyze biological samples to know the bioavailability, pharmacokinetics and/or metabolism of the compounds ingested by animal or human models in nutritional intervention studies. In addition, these studies have also been applied to determine changes of endogenous metabolites caused by prolonged intake of compounds with bioactive potential. This review aims to describe the main types and modes of application of high-resolution analytical techniques in all these steps for functional food development.

## 1. Introduction

In recent years, the interest in new food products (e.g., functional foods, nutraceuticals or foods supplements) has been increased by food industry. This kind of products is characterized by containing a high concentration of bioactive compounds which exert beneficial effects on human health. Secondary plant metabolites represent an important source of bioactive compounds used in the development of new functional ingredients in the agri-food and pharmaceutical industries. Phenolic compounds are the most characteristic example of bioactive compounds whose dietary intake has shown beneficial effects in different pathologies, such as obesity, cancer, neurodegenerative or cardiovascular diseases, among others [1,2,3,4].

For the functional food development, it is generally necessary to carry out several stages or sub-studies in order to obtain the desired product. Some of these stages are as follows: bioactive compound extraction, phytochemical characterization, compound isolation, bioactivity assays, encapsulation techniques, functional food formulation, industrial scaling, sensory evaluation and nutritional intervention assays [5,6,7]. Due to the fact that phytochemical concentrations in plant matrices are generally lower than the doses necessary to provide the beneficial effects, the purpose of the extraction step is the recovery of bioactive compounds from plant sources, achieving their isolating and concentration. The phytochemical characterization step aims to assign chemical identity and/or quantify concentrations of the compounds present in the plant matrices [8].

The development of a functional food is valueless if the phytochemicals are not stable in the food matrix or if they are not absorbed throughout the digestive system. For these reasons, the isolation/purification, the control of toxicity, the verification of the bioactivity properties and the determination of the bioavailability and metabolism of the characterized compounds, are also necessary stages for the development of functional products. In this context, encapsulation methodologies have been developed in order to protect the bioactive components in the food matrix as well as favor their absorption in the gastrointestinal tract [9,10,11]. Finally, once a functional food has been formulated, its sensory properties, the industrial scaling and the effect of its prolonged intake are studied before being placed on the market.

High-resolution analytical techniques play an important role in several of these stages of functional food development, especially phytochemical characterization of plant extracts [8] and the analysis of biological samples collected after nutritional intervention studies [12,13,14]. The objective of these studies is to identify the metabolites originated after the ingestion of certain compounds or foods that help to know the bioavailability as well as metabolic reactions that the original compounds undergo in the organism after being ingested. These studies are classified in acute and longitudinal intervention studies depending on whether biological samples are taken just after a single intake or if after consuming the product continuously over time.

The most used analytical platforms for these studies are nuclear magnetic resonance (NMR) and mass spectrometry (MS). NMR provides advantages in terms of structural elucidation and high precision in the quantification. In contrast, the main disadvantage of NMR is its lower sensitivity compared to MS [15,16]. On the other hand, MS stands out for its higher sensitivity and selectivity. Although there are applications that use MS by direct infusion, the great potential of this technique is achieved when it is previously coupled to a separation technique, such as liquid chromatography (LC), gas chromatography (GC) or capillary electrophoresis (CE), allowing the detection of hundreds of metabolites thanks to lower ionic suppression and reduced sample complexity [17,18,19]. This review aims to summarize the main modes of application of these analytical techniques in the different steps of the development of functional foods. Mainly, this review focuses on the application of these analytical techniques in phytochemical characterization and in the development of nutritional intervention studies.

## 2. Phytochemical Characterization of Bioactive Compounds

The characterization of complex extracts from natural sources consists in assigning the chemical structure as well as quantifying the concentration levels of the phytochemicals present in them. The exhaustive information of the phytochemical profile of natural matrices allows the discovery of new compounds, such as potential bioactive compounds or toxic compounds, providing a better knowledge of health effects and ensuring greater food safety, respectively. For that purpose, NMR, LC–MS and GC–MS have been the most used analytical platforms to achieve qualitative and quantitative characterization of plant extracts [19].

### 2.1. Mass Spectrometry (MS)

Mass spectrometry is based on the vacuum separation of ions in the gas phase according to their mass/charge ratio (*m*/*z*) [19]. It is important to note that there is a constant improvement in MS technology and new analytical approaches are being developed with access to numerous spectral databases. In detail, MS consists of three phases: ionization, mass analysis and detection. The molecular ions are produced in an ionization source. These ions are transferred to the mass analyzer, which separates them according to their *m*/*z* values. Then, the isolated ions reach the detector and finally the results are showed as a mass spectrum [20]. To this end, different mass analyzers can be employed such as single quadrupole (Q), triple quadrupole (QQQ), quadrupole ion trap (QIT), time of flight (TOF) and quadrupole-time of flight (QTOF). Among them, the high-resolution MS (HRMS) analyzers, such as TOF or orbitrap, have a greater applicability in characterization studies of bioactive compounds. The HRMS analyzers provide a precise relative quantification as well as exact mass values with an accuracy of less than 5 ppm. In addition, these analyzers also provide information about the isotopic distributions of chemical species, which is useful along with the exact mass data for the generation of molecular formulas that are useful to identify compounds [21]. On the other hand, tandem mass analyzers (MS/MS), such as QTOF, QIT or QQQ, provide fragmentation patterns of precursor ions that are of great application for metabolite identification and isomer resolution. As mentioned above, MS is usually coupled to separation techniques such as LC and GC with the purpose of improving the chemical characterization of phytochemicals contented in botanical sources. The following subsections detail the main applications of LC–MS and GC–MS in phytochemical characterization studies.

#### 2.1.1. GC–MS

Gas chromatography (GC) is an analytical technique whose objective is the separation of chemical compounds present in a complex matrix based on their volatilities and the interactions with a stationary column. GC–MS provides a robust analysis that allows the identification of analytes based on the comparison of the MS spectra with those available in GC–MS libraries. This hyphenated technique has been mainly used for characterization of low-molecular weight and medium or low polarity compounds, in particular, primary metabolites such as fatty acids, carbohydrates, amino acids and all volatile compounds [22].

A condition for GC analyses is that the analytes must be volatile enough to be able to be separated and detected. For this reason, many of the GC applications have been based on the characterization of the volatile compounds fractions of different plant or food matrices [23,24,25]. However, several non-volatile compounds can be derivatized prior to analysis in order to increase its volatility. Thus, a well-chosen derivatization procedure, based on the chemical composition of the target compounds, is crucial for improving the chemical separation, being the conversion of the analyte to its trimethylsilyl derivative, one of the most common derivatization methods [26,27]. This step is essential for non-volatile plant secondary metabolites such as phenolics, terpenoids, steroids and alkaloids.

Electron ionization (EI) and chemical ionization (CI) are the common ionization methods in GC–MS while Q, QQQ, TOF and Q-TOF are the most widely used mass analyzers. GC–TOF affords fast scanning, high sensitivity and mass accuracy compared to common quadrupole (GC-Q). However, GC–EI–Q–MS is the most widely used configuration due to its robustness, relatively low cost and the possibility of using libraries databases for compound identification, which is one of the main advantages of using an EI source [22,28]. Since EI is a hard ionization source, this platform offers robust information relative to fragmentation patterns of volatile compounds. The MS spectra are compared with the MS libraries, allowing the identification of the analytes such as phytosterols, terpenes or fatty acids that belongs to essential oils fraction of plants. Hence, numerous secondary metabolites from botanical samples have been identified and quantified by GC–MS. As shown in Table 1, GC–MS is able to determine the volatile fraction of plant sources.

#### 2.1.2. LC–MS

High performance liquid chromatography (HPLC) is a fast, versatile, reliable and efficient technique capable of separating components from a complex liquid mixture. LC involves a huge versatility regarding stationary and mobile phases [41]. Among the different LC configurations, reverse-phase (RP) mode has been the most used in the characterization of plant extracts. This RP-LC consists of a polar mobile phase and a hydrophobic stationary phase. Specifically, C_18_ columns are generally the most commonly used in this chromatographic mode. Different parameters (e.g., flow rate, type of column, particle size, mobile phase, gradient, temperature, etc.) have to be correctly optimized in an LC method to achieve a correct chemical separation. In recent years, ultra-high pressure liquid chromatography (UHPLC) is being used more for the analysis of complex samples, since it allows reaching higher pressures, allowing the use of columns with smaller particle sizes (<2 µm), and therefore achieving a higher chromatographic resolution [42,43].

LC–MS platforms are more suitable for characterization of non-volatile compounds with higher polarity and/or molecular weights. For example, flavonoids are generally present in their glycosylated form in plant sources, involving a high number of hydroxyl groups and higher molecular weights. Therefore, these intact glycosides are preferably analyzed by LC–MS [44].

The speed, sensitivity and resolution are improved in UHPLC methodologies. Another advantage of UHPLC is the significant reduction of analysis time, which also implies a reduction in solvent consumption [45]. Unlike GC, LC lacks a database library. Consequently, the identification of analytes is a more difficult task. In this sense, MS/MS analyzers, such QTOF, provide detailed information about characteristic fragments of bioactive compounds that facilitate the phytochemical characterization. The potential of these MS detectors covers the identification of a wide variety of polar and semi-polar compounds such as sugars, terpenes or phenolic compounds, among others [46]. Thus, as shows Table 2, LC coupled to different MS detectors is able to separate and to discern the phytochemical composition of botanical sources.

Despite the great advantages of MS coupled with chromatographic techniques, these methods still have limitations for obtaining the complete phytochemical characterization. These limitations are related to the resolution of isomers that present similar retention times and *m*/*z* ratios [47]. These limitations seem to have been overcome in recent years thanks to an additional coupling of ion mobility spectrometry (IMS) [48]. IMS has become the most valuable analytical technique for the analysis of complex mixtures, because it allows the discrimination of species that are neither separated by chromatography nor by MS. Thus, IMS is able to discriminate molecules with similar behaviors such as isomers, enantiomers, isobaric molecules and resolve isoforms and conformers. Therefore, the orthogonality of LC, IMS and MS provide instruments that, when combined, offer an enormous resolution capacity for increasing the level of coverage in the separation, identification of exact masses and quantification of components of complex samples such as botanical extracts. Although LC–IMS–MS is a recently emerging platform, some studies have already used it for plant extracts characterization. For instance, thirteen alkaloids from *Peganum harmal L.* or lignans from *Schisandra chinensis* oil were determined by HPLC–ESI–IMS–MS [49,50].

### 2.2. NMR

NMR was originally employed in the late 1940s to determine the structure of molecules in organic chemistry, but its application in food field was deferred until the 1980s. Nowadays, a large number of NMR-food studies have been developed such as food quality analysis, food composition or the metabolic profiling of medicinal plants [15,73,74,75].

Although NMR is not the best technique for phytochemical characterization due to its relatively low sensitivity compared to MS, it has also been used in various studies for the characterization of phytochemicals, such as phenolic compounds, in complex mixtures. For example, the polar fraction of virgin olive oil that contains several phenolic compounds was analyzed by this technique. The identification of the olive oil phenolic compounds was built on the chemical shifts of a large number of model compounds assigned by NMR. Thus, this methodology was successful in detecting simple phenols, such as vanillic acid, p-coumaric acid, vanillin, homovanillyl alcohol, free hydroxytyrosol and free tyrosol, the flavonols apigenin and luteolin, the lignans (+) 1-acetoxypinoresinol, (+) pinoresinol, (+)and syringaresinol, the dialdehydic form of oleuropein and ligstroside lacking a carboxymethyl group, two isomers of the aldehydic form of oleuropein and ligstroside, and total hydroxytyrosol and total tyrosol reflecting the total amounts of free and esterified hydroxytyrol and tyrosol, respectively [76]. In a more recent study, the polyphenols profile in olive oils was analyzed by Maximum-Quantum (MaxQ) NMR spectroscopy [77]. Three Italian olive oils were analyzed in which 24 compounds including organic phenols, lignans, secoiridoids and flavonols were identified. In addition, this approach managed to show phenolic compound profiles with significant differences in the three Italian olive oils analyzed.

NMR has been also applied to evaluate the composition changes of black garlic during a thermal treatment [78]. In this study, ^1^H NMR spectra showed that 38 compounds were altered by thermal procedure of raw garlic. Multivariate analysis reported changes in the contents of glucose, amino-acids, formic acid, fructose, acetic acid, cycloalliin, pyroglutamic acid, and 5-(hydroxymethyl)furfural. In another study, the green tea composition was analyzed during a fermentation processing by ^1^H NMR and multivariate statistical analysis [79]. 14 compounds including glucose, epigallocatechin, epicatechin, caffeine, alanine, epicatechin-3-gallate, theanine, acetate, quinate, glutamate, sucrose, or gallate were highlighted to be responsible for metabolic differentiation between green tea and fermented tea. For example, the levels of epigallocatechin, epicatechin, epicatechin-3-gallate, quinate, epigallocatechin-3-gallate, sucrose and caffeine decreased whereas glucose and gallate levels increased after this process. NMR technology has also been used to classify sample varieties according to their geographical origins. For instance, the chemical profile of white wines produced from “Greco bianco” grape variety in different Italian areas were determined by NMR [80].

One of the main advantages of NMR is its capacity for structural elucidation. This makes it a technique of special interest when identity cannot be characterized by MS, as in the case of isobaric compounds. For this reason, NMR has been applied for the analysis of previously isolated fractions using chromatographic techniques such as semi-preparative HPLC, column chromatography with silica gel, high speed counter-current chromatography (HSCCC) among others LC platforms [81,82,83]. For example, numerous studies use LC-NMR for the characterization of phenolic compounds. These examples include the identification of gallic acid, protocatechuic acids and anthocyanins from *Hibiscus sabdariffa* calyces [82,84], flavonoids from *Lippia gracilis* [85], procyanidins from cacao beans [86], epicatechin vanillate from grape seed and red wine [87], terpenoid glycosides from *Rosmarinus officinalis L*. [88], and flavonols and phenolic acids from red onion peels [89].

## 3. Nutritional Intervention Studies

Despite the fact that natural or dietary bioactive compounds receive enormous attention due to their potential health benefits, there is still a lack of knowledge in the assessment of their dietary intake. Although their bioactivities have been well-described by in vitro models, these effects verified by in vivo models are still limited. In this sense, inconsistent and low systemic exposure or poor oral bioavailability greatly limits the therapeutic uses of these bioactive compounds [90]. For this reason, it is necessary to develop accurate approaches that provide an understanding of the factors related to bioavailability. Consequently, bioavailability studies allow to know which bioactive compounds are able to reach the bloodstream and the target tissue, where they can exert their biological activities.

In recent years, studies on the metabolism of bioactive compounds have been increased considerably. However, data as doses, study organisms and time scales used in experimental studies are inherently difficult to search for and place into context. The nutritional intervention assays aim to know the effects on the organism of the consumption of a certain product (nutrient, bioactive compound, food extract, food, dietary supplement, diet, etc.). These types of studies are mainly classified into two groups depending on the time of the intervention. On the one hand, acute intervention trials investigate the effects of intake during the hours immediately after it, up to a maximum of 48 h. This group includes studies of absorption, pharmacokinetics, bioavailability or metabolism of ingested compounds. On the other hand, chronic or longitudinal nutritional intervention studies focus on the effect that the intake of that substance, food or diet under study causes in the body, prolonged over time, for weeks, months or years [91]. Moreover, the availability of “omics” technologies gives the possibility for exploring pharmacological actions of single and multi-compound formulations for the treatment of multifactorial disorders such as cancer, diabetes, cardiovascular and aging-associated diseases. To understand the impact of the bioactive compounds on human health, it is essential to establish the mechanisms providing the information about the factors that depend on their bioavailability after intake. These aspects are mentioned in the following subsections.

### 3.1. Acute Intervention Studies: Bioavailability and Pharmacokinetics

Bioavailability is defined as the fraction of an ingested ingredient that is bioaccesible, absorbed, and able to reach the circulatory system to be distributed to organs and target tissues where it can exert its health benefits. Several factors that affect the bioavailability of a certain compound have been described, such as dose, synergistic effects, matrix release and transport in the body or excretion. Since biological properties of dietary bioactive compounds depend on their bioavailability, different in vivo studies have been reported to measure their concentrations in plasma and urine after the intake of isolate bioactive compounds, extracts or foods [92,93].

Numerous human and animal models have been used to evaluate the pharmacokinetics and bioavailability of phenolic compounds. These types of studies have been carried out thanks to the potential of high-resolution analytical techniques. Given the high resolution and sensitivity of these techniques, there are many applications for the analysis of biological samples, allowing the identification and/or quantification of bioavailable compounds in low concentrations. Among them, LC–MS methods have been the most widely used for bioavailability and pharmacokinetic studies in recent years [66,94,95,96,97,98,99,100,101,102,103,104,105].

**Figure 1:**
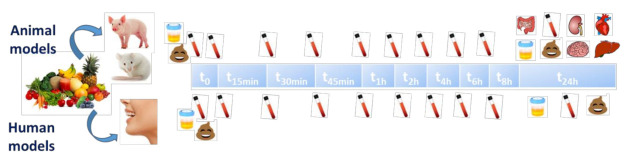
Scheme of a typical acute intervention study carried out in animal and human models.

For the development of functional foods, bioavailability studies should be ideally performed in vivo on humans, but this is not always ethically and financially possible. However, animal studies are necessary to provide knowledge about uptake and distribution of bioactive compounds in body tissues, which are important factors for the evaluation of the biological activity. Figure 1 shows the common procedure for acute intervention studies using animal or human models. Both types of assays are characterized by the collection of biological samples throughout the hours after the intake of the compounds of interest. The main difference between both assays lies in the major possibilities of biological sample collection in case of animal models. Regarding tissues, heart, liver, spleen, lung, kidney, stomach, intestine, brain, muscle, adipose, testis or ovary have been explored in different bioavailability studies in animal models [100,102,104].

Regarding the bioavailability of phenolic compounds determined by in vivo models, total polyphenol intake detected in biological samples has been only estimated at 1–10% being significantly low for flavones as quercetin and rutin (0.3–1.5%), but higher for flavanols (tea catechins), flavanones (naringenin from citrus fruits), soy isoflavones (genistein) and red wine anthocyanidins (3–30%) [106]. In this context, there are recent studies which use validated analytical methods to determine the bioavailability and pharmacokinetic parameters. For instance, Zeng et al. studied different parameters related to pharmacokinetics, tissue distribution, metabolism, and excretion of naringin in aged rats [100]. In this study, LC–QQQ–MS/MS method was applied for the quantification of naringin and naringenin in plasma, urine, feces, and various tissue samples. In addition, 39 flavonoid metabolites and 46 microbial-derived phenolic catabolites could be identified by UHPLC-Q-TOF analysis. They detected significant differences between the pharmacokinetic parameters between female and male aged rats. Tissue distribution profiles at different time points showed that the distribution of naringin and its metabolite after single oral dose was higher in gastrointestinal tract and major organs, with the major concentration at 3 h and 6 h, respectively [100]. Tao et al. developed a robust UHPLC–MS/MS method for the simultaneous determination in rat plasma of cinnamic acid, vanillic acid and protocatechuic acid from *Cinnamomum cassia.* They showed that the maximum concentration in plasma was obtained one hour after oral administration [96]. In addition, this approach was applied for the quantification of seven catechins in rat plasma [95]. This method was also applied by Chang et al. for the determination and quantification of mangiferin, single *Anemarrhenae Rhizoma* extract and multiple botanic extract of Suan-Zao-Ren decoction (SZRD) in rat plasma [107]. Zhang et al. also verified that this UHPLC–MS/MS method is suitable for the separation and simultaneous determination of two bioactive compounds (angoroside C and its metabolite ferulic acid) in biological samples from rats [104].

Nevertheless, a key element that must be taken into account by researchers is the concept of extrapolation of dose between species when new animal or human models are designed. Nair and Jacob et al. have tried to shed light through allometric scaling of basic information about dose conversions between species and estimation of starting dose for clinical trials [108]. Several authors aimed to expand the knowledge on the metabolic fate of dietary polyphenols such as sample preparation. This is a challenge since cell matrix and the hemoglobin content may have a significant influence. In this sense, Mülek and Högger established an efficient method for the quantification of selected polyphenols in human blood cells by modifying the principles of the QuEChERS approach, which also give valuable information about the in vivo distribution of bioactive polyphenols metabolites produced by gut microbiota [94].

It is well known that different bioactive compounds can undergo important transformations during their metabolization before reaching the target tissues. For example, these compounds may first be hydrolysed by gastric juice in the stomach. They can also be metabolized by intestinal cell enzymes or catabolized by the colonic microbiota, which can drastically affect the absorption of these molecules through the gut barrier. In fact, it is quite common that the corresponding aglycones compounds are generated through enzymatic hydrolysis reactions. Dietary phenolic compounds can also undergo phase II metabolism reactions in the small intestine by enzymes such as β-glucosidases, UDP-glucuronosyltransferase or catechol-O-methyltransferase. Additionally, once the compounds or derived metabolites have passed into the bloodstream, they may continue to undergo phase I and II metabolic reactions in the liver, such as oxidation-reduction, methylation, sulfation or glucuronidation reactions [109,110,111]. The detection of possible derivative metabolites in biological samples has been possible thanks to the advances of high-resolution analytical techniques and the field of metabolomics. For instance, Achour et al. recently identified phase II metabolites from rosemary in plasma and urine samples using a targeted metabolomic approach based on HPLC–MS–QTOF [112]. In another study, Gómez-Juaristi et al. used a similar methodology to identify phase II metabolites from cocoa in a dietary intervention carried out in healthy humans [113].

Moreover, recent studies in human about pharmacokinetic profiles of different polyphenols provide valuable information to design future clinical interventions studies. This is crucial above all compounds with extensive degradation and metabolism. In this scenario, de Ferrars et al. have used a ^13^C-labelled anthocyanin and isotope-ratio MS in order to study the absorption and metabolism of these compounds. In this study, 17 circulating blood metabolites were detected and, on the other hand, 31 and 28 metabolites were found in the urine and feces, respectively [98]. However, the major limitation of this analytical methodology was the high number of compounds that were not identified (unknowns). For this reason, additional synthesis of the speculated metabolites or alternative analytical techniques such as GC–MS/MS and HPLC–TOF–MS would enable to identify further metabolites not captured by this methodology [98].

### 3.2. Longitudinal Intervention Studies

Longitudinal nutritional intervention studies are based on the observation and detection of physiological changes due to prolonged intake, for weeks or months, of a given daily dose of a food, compound, extract or diet. These types of trials can be applied with different objectives, such as studying the influence that a certain diet has on the risk factors of different pathologies (e.g., cardiovascular diseases, diabetes, cancer, etc.) [114]; or determining the impact of the consumption of certain foods, bioactive compounds or nutrients on the metabolic pathways [115,116]. Numerous longitudinal studies have been performed focused on anthropometric measurements (weight, height, waist and hip circumference) or in simple measurements in blood such as fasting blood glucose, insulin sensitivity or global lipid profiles (triglycerides (TG), low-density lipoprotein cholesterol (LDL-C), high-density lipoprotein cholesterol (HDL-C) or total cholesterol (TC)) [117,118].

**Figure 2:**
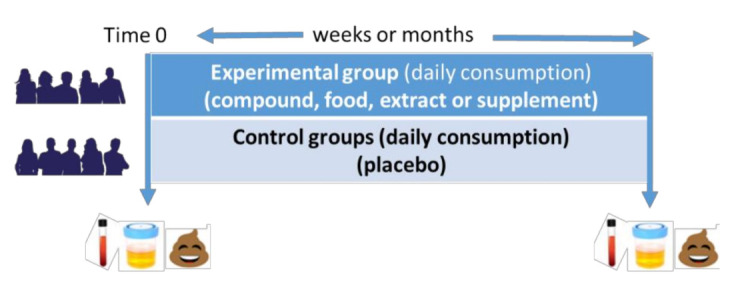
Scheme of a typical longitudinal intervention study carried out in humans.

In the same way as acute intervention studies, the longitudinal studies can be carried out in both animal and human models, where biological samples are usually collected at the beginning and at the end of the clinical trial for the identification of altered endogenous metabolites after nutritional intervention (Figure 2). Participants must be selected through previously established inclusion and exclusion criteria, since numerous factors can affect the results of the study, such as age, gender, lifestyle, health status, status metabolic, etc. To verify the results obtained and guarantee their quality, the use of randomized double-blind studies is required through the use of control groups that consume a placebo diet, in which participants do not know their membership in the assigned experimental group [91].

Unlike acute intervention studies, the use of high-resolution techniques in this type of study is focused on knowing the alterations produced in endogenous metabolites due to intake instead of detecting the original compounds or their related metabolites in biological samples. In this context, the emergence in the last decade of the metabolomics field and the high-resolution analytical techniques, has allowed the application of these tools in longitudinal nutritional intervention assays in order to know the impact on the endogenous metabolic pathways produced by a certain intake. For longitudinal nutritional studies, these analytical techniques are typically applied within the workflow of targeted and untargeted metabolomic strategies. Targeted strategies focus on evaluating how the intake of a certain potential bioactive ingredient or functional food affects specific metabolic pathways. The selection of the target metabolites or metabolic pathways is normally related to the beneficial effects that are intended to be demonstrated or based on commercial kits [91,119].

The metabolomic untargeted strategies are based on detecting the greatest possible number of meaningful signals in the biological samples. It is important to note that no technique is able to analyze the entire metabolome. For instance, in LC–ESI–MS, reverse phase (RP) mode allows to separate semipolar compounds such as lipids, vitamins, alkaloids, glycosylated steroids and other glycosylated species. On the other hand, hydrophilic interaction chromatography (HILIC), which is gaining popularity in recent years, is capable of separating the most polar compounds such as sugars, amino sugars, amino acids, vitamins, carboxylic acids or nucleotides, among others [120]. Regarding GC–MS, this hyphenated technique is used for the analyses of low molecular weight volatile metabolites, and non-volatiles after derivatization steps (amino acids, organic acids, sugars, amines, alcohols, amides) [121].

Despite the lower sensitivity of NMR compared to MS, NMR is also being applied in metabolomic studies in recent years. This technique has multiple advantages such as its non-destructive, nonbiased, high reproducibility, little sample treatment and greater capacity for large-scale metabolomic studies. In addition to these advantages, NMR is especially sensitive to analyzing compounds that are more difficult to detect with LC–MS, such as alcohols, organic acids, sugars, polyols, and other highly polar compounds. [122].

The majority of longitudinal nutritional intervention studies has been highly applied using foods that are consumed daily in the diet such as cheese, milk, vitamins, butter or fish oils, among others [115,123]. Due to the development of new products in recent years, such as nutraceuticals of functional foods, this type of study is currently being applied to bioactive compounds, plant extracts or dietary supplements with the aim of knowing their health effects in the metabolism after its prolonged intake [116,124]. Table 3 lists a representative number of longitudinal nutritional intervention studies conducted with metabolomic methodologies. These types of studies have been carried out in order to detect the metabolic changes produced by an isolated bioactive compound, such as vitamin E [124], catechin [125] or resveratrol [126]; bioactive extracts (curcuma [127], rosemary [128], grape skin [129], etc.); or functional supplements based on garlic [130] or grape skin polyphenols [116], among others. The different studies carried out with grape extracts or supplements are a clear example of the complementarity of the different analytical techniques to study the different compound classes of the metabolome due to the fact that these studies have been conducted using NMR [131], LC–MS [116] or GC–MS [132] techniques.

Furthermore, these studies can be divided into two types depending on whether a model of healthy individuals or a specific pathology is used. In this sense, longitudinal studies have been applied to determine how the consumption of certain bioactive products affects the metabolism of different pathologies such as metabolic syndrome [126], hypercholesterolemia [133], diabetes [128], irritable bowel syndrome [134] or cardiovascular diseases [135], among others.

In addition to the importance of the aforementioned analytical techniques, other steps are required in longitudinal nutritional studied based on untargeted metabolomic methodologies such as data processing, statistical, metabolite identification and pathway analysis techniques. Given the high number of signals obtained in the analyses by MS or NMR, sophisticated data processing techniques must be carried out, encompassing peak picking, alignment, normalization or annotation techniques, among others [136,137]. Once the data is processed, the statistical techniques are applied to select the variables that show a significant effect after the intake of the product studied. These techniques are classified, in univariate analyses (e.g., *t*-test, ANOVA, fold change, etc.) and multivariate analyses such as principal component analyses (PCA), partial least squares–discriminant analysis (PLS-DA), orthogonal projections to latent structures discriminant analysis (OPLS-DA), random forest (RF) or hierarchical cluster analysis (HCA) [138]. Univariate methods are characterized by the analysis of each variable individually and independently while multivariate methods take into account all the variables simultaneously, making it possible to identify relationships and interactions between the different metabolites [138]. Given the high number of variables detected in this type of studies, there is a high probability of finding false negative and/or false positive results in statistical models. Therefore, the validation of statistical models is a necessary step to verify and guarantee the quality of the results. In this sense, there are mainly three validation methods: cross-validation (CV), permutation analyses and external validation. CV methods are based on the division of the data set into two subsets for use in the construction and validation of the models, separately. In contrast, the external validation uses a new set of samples for this purpose. Regarding permutation test, this is used in order to verify that the statistical model does not present over-fitting. This is based on using the classification model with the classes randomly assigned. In this way, the results obtained by the original model are statistically compared with those obtained by means of permutations [139].

Finally, the significant signals obtained in the statistical analyses must be identified in their corresponding endogenous (or exogenous) metabolites. Whereas NMR has the potential to assign chemical structures, MS studies presents greater limitations for the identification of metabolites. In MS studies, GC-EI–MS has greater advantages for identification because there are chemical libraries that facilitate the identification of metabolites by comparison of the fragments obtained after electron ionization. On the other hand, LC–MS studies needs to perform tandem mass spectrometry analysis (MS/MS) to obtain characteristic fragments of the metabolites to be compared with the information available in different databases, such as HMDB, KEGG, LipidMaps or METLIN [140]. In spite of these tools, the identification step remains a bottleneck in untargeted metabolomic studies based on MS since many of the signals cannot be correctly identified [141].

The application of metabolomics and analytical techniques in longitudinal intervention studies provides knowledge about the effects of prolonged ingestion of a compound with potential bioactivity on metabolism. This knowledge can be essential to better understand the bioactive effects and have more information to support the use of these compounds for the development of functional foods. Despite this potential application of analytical techniques and untargeted metabolomics in longitudinal intervention studies, there are still not many studies carried out with functional foods. Therefore, this type of study may have great potential for development in the coming years in the field of functional food research.

## 4. Conclusions

The rapid evolution of high-resolution analytical techniques such as LC–MS, GC–MS and RMN, has contributed to an enormous improvement in this field of food science. These techniques have proven to be fundamental tools at various steps of functional food development. These have been used in numerous phytochemical characterization studies as well as in the analysis of biological samples collected in bioavailability, metabolism and longitudinal intervention studies. The use of analytical platforms is also useful for the discovery of new biomarkers in the field of food science field and biomedicine, as they offer detailed and reliable information about the metabolism of bioactive compounds, including their bioavailability and pharmacokinetics. These results help in a considerable extent in the functional food or nutraceutical development with bioactive effects in different disorders. In spite of the high sensitivity of these analytical platforms, further upgrades are needed to increase the ability to resolve isomers or discern a larger number of metabolites generated, including those that are below the detection limits. Promising advances in these techniques, such as the use of IMS coupled to LC–MS, will allow an advance in knowledge in the field of functional food development in the coming years.

## Figures and Tables

**Table 1:** Applications of GC–MS to characterize bioactive compound from natural sources.

MS Detector	GC Column	Gradient (°C)	Compounds Detected	Matrix	Ref.
TOF	DB-5 MS (30 m)	60 to 300 (multistep increase)	Terpenes	*Curcuma species*	[29]
	BPX5 (30 m)	40 to 270 (4 °C/min)	Essential oils	*Rosmarinus officinalis*	[30]
Q	RTS volatile (30 m)	70 to 280 (5 °C/min)	Organic sulfur compounds	*Allium sativum*	[31]
	HP-88 (100m)	70 to 230 (10 °C/min)	Essential oils	*Lippia citriodora*	[32]
	RESTEK Rtx^®^-5 (30 m)	70 to 320 (7 °C/min)	Essential oils and derivatives	*Allium cepa*	[33]
	SUPELCOWAX 10 (30 m)	60 to 240 (4 °C/min) 40 to 240 (multistep increase)	Essential oils	*Camellia sinensis*	[34]
	HP-5MS (30 m)	50 to 280 (multistep increase)	Essential oils	*Curcuma species*	[35]
	DB-1MS (60 m)	50 to 180 (2 °C/min)	Essential oils and hydrosols	*Rosmarinus officinalis*	[36]
	SUPELCOWAX 10 (30 m)	50 to 200 (5 °C/min)	Essential oils	*Rosmarinus officinalis*	[37]
QQQ	HP-5MS (30 m)	80 to 300 (multistep increase)	Essential oils	*Curcuma longa*	[38]
Q-TOF	HP-5MS (30 m)	250 to 310 (multistep increase)	Triterpenoids	*Olea europaea*	[39]
	HP-5MS (30 m)	250 to 310 (10 °C/min)	Phytosterols and Tocopherols	*Mangifera species*	[40]
	RTX-200MS (30 m)	250 to 310 (10 °C/min)			

**Table 2:** Applications of LC to characterize bioactive compound from natural sources.

LC Platform	MS Detector	Column	Ionization Mode	Compounds Detected	Matrix	Ref.
HPLC	TOF	Zorbax Eclipse Plus C18	Negative	Organic acids, phenolic acids and flavonoids	*Hibiscus sabdariffa*	[51]
		RP18	Negative	Flavonoids, terpenoids	*Rosemary officinalis*	[52]
		Zorbax Eclipse Plus C18	Negative	Iridoid glycosides, flavonoids and phenylethanoids	*Lippia ciriodora*	[53]
	Quadrupole	Eclipse XDB C_18_	Positive	Anthocyanins	*Allium cepa*	[54]
		Phenomenex Luna C_18_	Negative	Phenolic acids and flavonoids	*Curcuma longa*	[55]
	Triple Quad	Zorbax Eclipse XDB-C_18_	Negative	Phenolic compounds	*Trametes versicolor*	[56]
	Q-TOF	YMC C_30_ Synergi Hydro-RPC_18_	Positive and negative	Carotenoids, organic acids, phenolic acids and flavonoids	*Hibiscus sabdariffa*	[57]
		Zorbax Eclipse Plus C18	Negative	Flavonoids	*Theobroma cacao*	[58]
	Orbitrap	Zorbax Eclipse XDB-C_18_	Positive and negative	Flavonoids	*Allium ampeloprasum*	[59]
		Zorbax Eclipse Plus C_18_	Negative	Secoiridoids, flavonoids and related compounds	*Olea europaea*	[60]
	Qtrap	Waters Spherisorb S3 ODS-2 C_18_	Negative	Phenolic acids and flavonoids	*Rosemary officinalis*	[61]
UHPLC	TOF	Acquity UHPLC BEH C_18_	Positive and negative	phenolic acids flavonoids and gallotanins	*Camellia sinensis*	[62]
	Quadrupole	Acquity UHPLC BEH C_18_	Positive	Valifenalate	*Vitis vinifera*	[63]
		Acquity HSS T3	Positive and negative	Hydroxycinnamic acids	*Vitis vinifera*	[64]
		Acquity UPLC shield RP-18	Negative	Epigallocatechin	*Camellia sinensis*	[65]
	Q-TOF	Waters ACQUITY UPLC BEH C18	Negative	Phenolic acids, flavonoids	*Glechoma longituba*	[66]
		Waters HSS-T3 column Waters C_18_ BEH Waters BEH-Amide	Positive and negative	Phenolic acids, anthocyanins and organosulfur compounds	*Allium sativum*	[67]
		ACQUITY UPLC HSS T3	Negative	Procyanidin	*Theobroma cacao*	[68]
		Hypersil Gold	Negative	Terpenes, flavonoids, phenolic acids	*Rosemary officinalis*	[69]
		Waters Acquity UPLC C_8_	Positive and negative	Epigallocatechin gallates	*Camellia sinensis*	[70]
	Orbitrap	Phenomenex Luna C_18_	Positive and negative	Phenolic acids, flavonoids, tannins and anthocyanin-derived pigments	*Punica granatum*	[71]
		Zorbax SB-C_18_	Negative	Stilbenes	*Vitis Vinifera*	[72]

**Table 3:** Application of analytical approaches in longitudinal nutritional studies.

Bioactive Compound, Extract or Supplement	Analytical Technique	Biological Samples	Population	Dose/Intervention	Results	Ref
Catechin	LC(RP)-QTOF–MS	Plasma, urine, heart, liver and aorta	24 male Wistar rats divided in different groups	Diet supplemented or not with 0.2% (+)-catechin.	76 variables affected by catechin supplementation	[125]
Resveratrol	UPLC(RP)-Orbitrap–MS/MS GC-EI-Q–MS	Blood, urine, adipose tissue, skeletal muscle tissue	66 men subjects with metabolic syndrome	(a) 75 mg twice daily (b) 500 mg twice daily (c) Placebo Duration: 4 months	Reduced sulphated androgen precursors, long-chain PUFAs (n-3 and n-6) increased in adipose tissue,	[126]
Vitamin E	LC(RP)–MS(QTOF)	Plasma samples	10 male adults	400 mg/d of RRR-α-tocopheryl acetate (4 weeks)	Influence in phospholipid metabolism and lysoPC generation	[124]
Cocoa (*Theobroma cacao* L.)	LC(RP)-QQQ–MS/MS	24 h urine and fasting plasma samples	19 men and 23 women presenting high risk of cardiovascular heart disease	(a) 20 g/day of cocoa powder with 250 mL skimmed milk (b) Placebo (500 mL/day of skimmed milk) Duration: 4 weeks	Increase in the urinary excretion of colonic microbial-derived phenolic metabolites, and biomarkers of the intake.	[135]
Chamomile extract	^1^H NMR	Urine samples	14 healthy volunteers	5 g of powder in 200 mL of boiling water (5 times/day). Duration: 2 weeks	Depletion of creatinine and the elevation of hippurate, glycine	[142]
Curcuma longa extract (1% of curcuminoids)	^1^H NMR, GC-ITQ–MS (Fatty acids)	Serum samples	30 rats (animal model)	(a) HFS diet with a Curcuma longa extract. (b) HFS diet (control) (c) standard diet Duration: 10 weeks	Metabolic differences in MUFA, n-3 PUFA, glycoproteins, glutamine and methanol (fatty acid metabolism)	[127]
Rosemary extract	LC(RP)-QTOF–MS	Urine samples	30 streptozotocin-induced diabetic rats	R.officinalis (150 mg) plus folic acid (15 mg)	Several amino acids and their metabolites point to changes due to the effect of the gut microbiota	[128]
Soy	GC-Q–MS	Fecal samples	35 female rats	SOY diet (590 mg/kg od soy isoflavones) or placebo. Duration: 29 weeks	SOY-fed animals had greater fecal concentrations of the beneficial bacterial metabolite, S-equol	[143]
Soy beef and grapefruit juice	^1^H NMR	Serum samples	25 healthy male volunteers	140 g of soy bean beefs and 500 mL of grapefruit juice per day for four consecutive days	Decrease of lactate, cholesterols and triglycerides	[131]
Probiotic fermented milk	^1^H NMR	Serum and fecal samples	74 patients with irritable bowel syndrome	Probiotic fermented milk (150 mL) or placebo 3/times daily for 8 weeks	Dysregulation in energy homeostasis (serum glucose) and liver function (serum tyrosine)	[134]
Supplement based on polyphenols from wine and red grape	GC-TOF–MS	Blood, 24 h urine, and fecal samples	26 adult human volunteers	Cellulose capsules with a polyphenol-rich mix of red wine and red grape juice extracts (800 mg of polyphenols) or placebo. Duration: 4 weeks	Increase of phenolic acids and evaluation of the gut microbial impact	[132]
Functional food based on grape skin polyphenols	LC(RP)-QTOF–MS	24 h urine samples	31 volunteers	187 mL of the functional beverage, containing a grape skin extract Control beverage as a placebo Duration: 15 days	Presence offirst-stage microbial metabolites of flavanols Several epicatechin and phenolic acid metabolites as markers of the intake	[116]
Red wine and grape juice extracts (WGM)	GC-Q–MS LC(RP)-QQQ–MS	Urine and plasma samples	35 healthy males	WGM in gelatin capusles (870 mg of dry weight red wine extract and 540 mg of dry weight grape juice extract) or placebo. Duration: 5 days	Alteration of microbial protein fermentation and amino acid metabolism (tyrosine, tryptophan).	[129]
Garlic (allium sativum) supplement Aliocare^®^	LC(RP)-ESI-QTOF–MS	Plasma samples	30 healthy volunteers (15 men and 15 women)	One gelatine capsule contained per day (70 mg of garlic supplement). Duration: 1 month	Increase in lysophosphatidylcholines, lysophosphatidylethanolamines and acylcarnitines. Decrease of four fructosamines	[130]

## Data Availability

Not applicable.
